# ESI-IMS–MS: A method for rapid analysis of protein aggregation and its inhibition by small molecules

**DOI:** 10.1016/j.ymeth.2015.05.017

**Published:** 2016-02-15

**Authors:** Lydia M. Young, Janet C. Saunders, Rachel A. Mahood, Charlotte H. Revill, Richard J. Foster, Alison E. Ashcroft, Sheena E. Radford

**Affiliations:** a Astbury Centre for Structural Molecular Biology, University of Leeds, Leeds LS2 9JT, United Kingdom; b School of Molecular and Cellular Biology, University of Leeds, LS2 9JT, United Kingdom; c School of Chemistry, University of Leeds, LS2 9JT, United Kingdom

**Keywords:** Aβ, amyloid-β peptide, Aβ40, amyloid-β peptide residues 1–40, AD, Alzheimer’s disease, ADH, alcohol dehydrogenase, AFM, atomic force microscopy, CCS, collision-cross sectional area, Cl-NQTrp, chloronaphthoquinine-tryptophan, CsI, caesium iodide, DMSO, dimethyl sulfoxide, EGCG, (−)-epigallocatechin gallate, ESI-IMS–MS, electrospray ionisation-ion mobility spectrometry–mass spectrometry, HDMS, high-definition mass spectrometry, hIAPP, human islet amyloid polypeptide, HTS, high-throughput screen, *m*/*z*, mass to charge ratio, ROCS, Rapid Overlay of Chemical Structures, (T)EM, (transmission) electron microscopy, ESI-IMS–MS, Amyloid, Small molecule inhibitor, Ligand screening, Aβ

## Abstract

Electrospray ionisation-ion mobility spectrometry–mass spectrometry (ESI-IMS–MS) is a powerful method for the study of conformational changes in protein complexes, including oligomeric species populated during protein self-aggregation into amyloid fibrils. Information on the mass, stability, cross-sectional area and ligand binding capability of each transiently populated intermediate, present in the heterogeneous mixture of assembling species, can be determined individually in a single experiment in real-time. Determining the structural characterisation of oligomeric species and alterations in self-assembly pathways observed in the presence of small molecule inhibitors is of great importance, given the urgent demand for effective therapeutics. Recent studies have demonstrated the capability of ESI-IMS–MS to identify small molecule modulators of amyloid assembly and to determine the mechanism by which they interact (positive, negative, non-specific binding, or colloidal) in a high-throughput format. Here, we demonstrate these advances using self-assembly of Aβ40 as an example, and reveal two new inhibitors of Aβ40 fibrillation.

## Highlights

• Identification of small molecule inhibitors of protein aggregation using ESI-IMS–MS.
• Characterisation of binding mode as negative, specific, non-specific or colloidal.
• High throughput ligand screening using ESI-IMS–MS.
• Identification of novel small molecule inhibitors of Aβ40 aggregation.

## Graphical abstract

**Figure:**
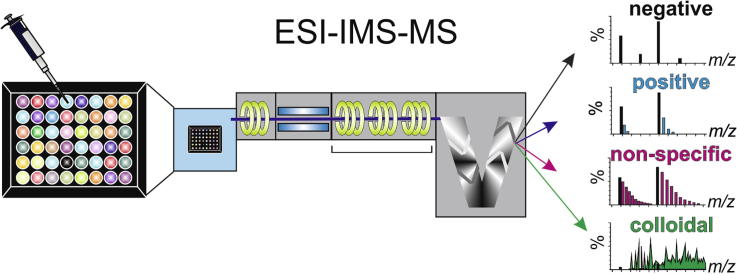

## 1 Introduction

Amyloidosis contributes to more than 50 human disorders including Alzheimer’s disease (AD) [1], the most common form of dementia worldwide [2]. The accumulation of the amyloid-β peptide (Aβ) in extracellular plaques in the form of highly ordered amyloid fibrils is a hallmark of AD, but it is the pre-fibrillar oligomers that are thought to be the major neurotoxic species [3]. Due to the complex mechanisms involved in AD and other amyloid diseases, there are currently few therapies available. Indeed, as the toxic species in many of these disorders remain elusive, current therapies focus on ameliorating symptoms, rather than preventing disease progression [4]. The identification and characterisation of the potentially toxic oligomers populated *en route* to amyloid fibrils is a significant challenge due to the heterogeneous, transient and lowly-populated nature of these species. ESI-IMS–MS has the unrivalled ability to study such systems given its unique potential to detect and identify multiple ions present at low concentrations within the same sample, based on their mass-to-charge ratio (*m*/*z*) [5], [6], [7], [8], [9]. When coupled to IMS, further separation of ions of the same *m*/*z* ratio but different collision-cross sectional areas (CCS) is enabled, allowing different conformational states of isobaric protein oligomers to be characterised simultaneously [5], [9], [10], [11], [12], [13], [14]. Changes in protein conformation, and appearance and subsequent disappearance of oligomeric states, can be monitored over time [5], [13], [15], [16], [17]. Furthermore, as native ESI-IMS–MS allows the preservation of protein-ligand complexes, the binding interactions of small molecules to amyloid peptides/proteins can be observed, concomitant with changes in the relative abundances and distributions of oligomeric species present. These changes can then be correlated to alterations in fibril formation rate or yield [5], [7], [9], [17], [18], [19], [20] allowing identification of novel inhibitory compounds. The specific conformational states to which inhibitors bind can also be determined [5], [13], [17], and the mode of inhibition can be elucidated by simple analysis of the resulting spectra [18].

**Fig. 1:**
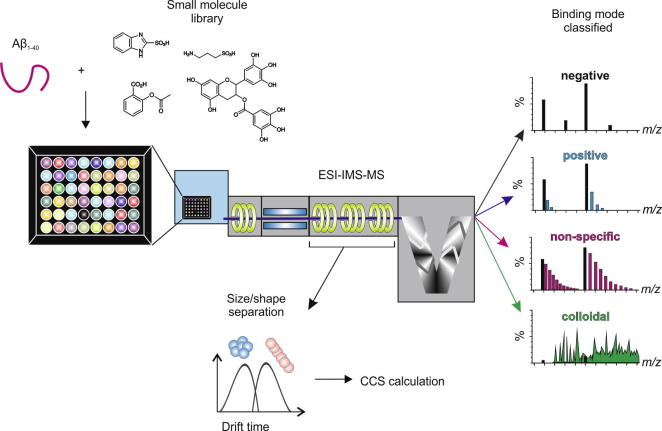
Schematic of the ESI-IMS–MS experimental procedure. The protein of interest is mixed individually with small molecules from a compound library in 96-well plate format. Via a Triversa NanoMate automated nano-ESI interface, the samples are infused into the mass spectrometer, wherein separation occurs based on the mass to charge ratio (*m*/*z*) and collisional cross-sectional area (CCS). A non-interacting small molecule will produce a spectrum the same as that generated by the peptide alone (black). A small molecule that specifically interacts with the peptide will produce a binomial distribution of bound peaks (blue) [45]. A non-specific ligand will bind but result in a Poisson distribution of bound peaks (pink) [45]. A colloidal inhibitor will produce a range of overlapping peaks due to self-association of the small molecule (green).

Here we demonstrate the power of ESI-IMS–MS as a method able to provide rapid and accurate analysis of protein aggregation and its inhibition, using self-assembly of Aβ40 into amyloid fibrils as an example system. The basis of the experimental set up is shown in Fig. 1. A further example, using amylin involved in type II diabetes mellitus, can be found in Young et al. [18].

## 2 Methods

### 2.1 Sample consideration

The most important parameter to consider in sample preparation for analysis by ESI–MS is the buffer in which the aggregation process is to be studied. Most *in vitro* biochemical techniques used to study amyloid assembly utilise involatile buffers that are incompatible with ESI–MS. This leads to issues with efficient ionisation of the sample and extensive adduct formation [13], reducing the quality of the resulting spectra. It is necessary, therefore, to conduct MS experiments in aqueous, volatile buffers such as ammonium acetate, ammonium formate or ammonium bicarbonate. *Note:* Simply replacing a non-volatile buffer with an MS-compatible buffer at the same pH and ionic strength may not yield the same rate of, and/or products of, aggregation. Ion composition, as well as ionic strength and pH, can influence aggregation parameters. We suggest, therefore, that the aggregation process under these conditions should be characterised prior to analysis by ESI–MS, using solution assays (e.g. dye binding assays, light scattering, or imaging of aggregates via electron microscopy (EM)/atomic force microscopy (AFM) (reviewed in [21])), to confirm that the assembly mechanism is similar in the non-volatile and ESI–MS-compatible buffers of equivalent ionic strength and pH.

Proteins stored or purified in non-volatile buffers, such as Tris·HCl, should be stringently buffer-exchanged, and concentrated if necessary, prior to analysis by ESI–MS. Working protein concentrations of low micromolar range are typical.

### 2.2 Sample and small molecule preparation

For the current study, an ESI-IMS–MS screen of the interactions of small molecules with Aβ40 at pH 6.8 was undertaken.

1. Aβ40 was expressed recombinantly and purified as described previously [18], [22]. *Note:* Synthetic peptide could be used in place of recombinant peptide [6], [9], which yields similar results (data not shown). However many preparations contain impurities that may complicate MS-based analyses and affect aggregation [23]. Therefore, care should be taken in ensuring sufficient sample clean-up.
2. Importantly, in the context of this screen, the final stages of purification involved size exclusion chromatography (Superdex™ 75 GL 10/300 column, GE Healthcare, UK) with a volatile mobile phase (50 mM ammonium bicarbonate, pH 7.8) and peptide-containing fractions were lyophilised. This step yields pure peptide, free from buffer salts, which can be diluted directly into MS compatible buffers and therefore requires no further buffer exchange. Pure recombinant Aβ40 peptide (containing an additional N-terminal methionine not present in wild-type Aβ40 produced by the cleavage of amyloid precursor protein) was then resolubilised in DMSO at 3.2 mM and diluted into 200 mM ammonium acetate, pH 6.8, 1% (*v*/*v*) DMSO at a final peptide concentration of 32 μM. The sample was centrifuged at 13,000*g* (4 °C, 10 min) prior to MS analysis to remove any insoluble aggregates that may have formed.
3. Caesium iodide solution, for mass calibration, was prepared by dissolving the compound in 50% (*v*/*v*) water/isopropanol to a concentration of 2 mg/mL.
4. The small molecules selected for screening for binding to Aβ40 were prepared freshly on the day of analysis. Compounds were solubilised in the relevant solvent (H_2_O, ethanol or DMSO) to create stock solutions of 10 mM small molecule. The solvent chosen must solubilise the compounds completely at room temperature.
5. In a 96-well plate format, small molecules (final concentration of 320 μM) were added individually to Aβ40 to give a molar ratio of 1:10 peptide to small molecule. The distribution of monomer, monomer-ligand and oligomer species populated in the presence of each small molecule was analysed immediately (within 2 min of addition, at room temperature), using ESI-IMS–MS.

### 2.3 ESI-(IMS)–MS

A Synapt HDMS quadrupole-time-of-flight mass spectrometer (Waters Corpn., Wilmslow, Manchester, UK), equipped with a Triversa NanoMate (Advion Biosciences, Ithaca, NY, USA) automated nano-ESI interface, was used for these analyses. The instrument has a travelling-wave IMS device situated between the quadrupole and the time-of-flight analysers (Fig. 1). The instrument has been described in detail elsewhere [24].

Aβ40 samples were analysed using positive mode nanoESI (nESI) with a capillary voltage of 1.7 kV and a nitrogen nebulising gas pressure of 0.8 psi. The following instrumental parameters were used: cone voltage 30 V; source temperature 60 °C; backing pressure 1.6 mBar; ramped travelling wave height 7–20 V; travelling wave speed 300 m/s; IMS nitrogen gas flow 20 mL/min; IMS cell pressure 0.55 mBar. Data were acquired over the range *m*/*z* 200–6000. Data were processed by use of MassLynx v4.1 and Driftscope software supplied with the mass spectrometer. The *m*/*z* scale was calibrated with aq. CsI cluster ions.

CCS measurements were estimated by use of a calibration obtained by analysis of denatured proteins (cytochrome c, ubiquitin, lysozyme) and peptides (tryptic digests of alcohol dehydrogenase (ADH) and cytochrome c) with known CCSs obtained elsewhere from drift tube ion mobility measurements [25]. Isotropic, linear and spherical oligomer growth models were estimated by the use of relevant equations. In isotropic growth, *σ_n_ = σ_monomer_* * *n*^2/3^, where *n* = oligomer number, *σ_n_* is the CCS of the oligomer number *n* and *σ_monomer_* is the monomer CCS [8]. Linear growth in one direction can be estimated by *σ_n_* = *a* * *n* + *k*, where *a* describes the CCS of a monomer within a fibril and *k* is the size of the fibril cap. In the spherical growth model, a spherical oligomer shape is assumed and expected CCSs are calculated based on a typical density *ρ* (in Da/Å^3^) of proteins and their complexes under comparable conditions [26]. Here, CCSs were calculated for a range molecular weights assuming a perfect sphere of density 0.44 Da/Å^3^ [12].

### 2.4 Transmission electron microscopy (TEM)

The TEM images of each peptide or peptide: ligand solution were acquired after 5 days incubation at 25 °C in low binding tubes (MAXYMum Recovery™ tubes, Axygen), using a JEM-1400 (JEOL Ltd., Tokyo, Japan) transmission electron microscope. Carbon grids were prepared by irradiation under UV light for 30 min and stained with 4% (*w*/*v*) uranyl acetate solution as described previously [27].

## 3 Results

### 3.1 Aβ40 forms an array of oligomers early in amyloid assembly

**Fig. 2:**
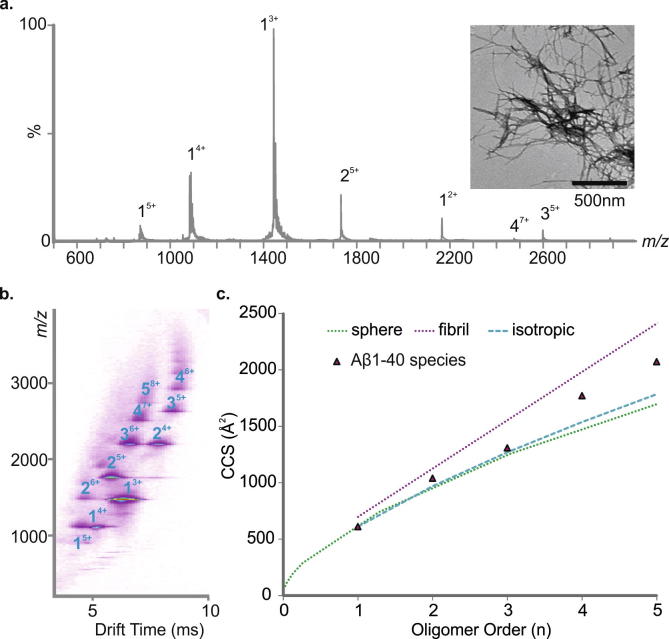
Analysis of Aβ40 oligomer distribution and collision-cross section (CCS). (a) ESI–MS mass spectrum of Aβ40. Numbers above peaks denote oligomer order, with the positive charge state of ions in superscript. Inset: negative stain TEM image of Aβ40 fibrils after 5 days in 200 mM ammonium acetate buffer, pH 6.8 (25 °C, quiescent) (scale bar = 500 nm). (b) ESI-IMS–MS Driftscope plot of the Aβ40 oligomers present 2 min after diluting the monomer to a final peptide concentration of 32 μM in 200 mM ammonium acetate, pH 6.8, 25 °C. ESI-IMS–MS Driftscope plots show IMS drift time versus *m*/*z* versus intensity (*z* = square root scale); (c) CCSs of Aβ40 oligomers measured using ESI-IMS–MS versus oligomer order; the CCS of the lowest charge state of each oligomer is shown (black triangles). The green dashed line represents a fit based on globular oligomers and the average density of a protein (0.44 Da/Å^3^) [28], the purple dashed line represents a linear growth model [8] and the blue dashed line represents an isotropic growth model [8].

Prior to performing inhibition studies, information regarding the aggregation process (number, identity and timescale of oligomer formation) of amyloidogenic proteins is required. To achieve this, oligomeric intermediates need to be identified and characterised. Here, by exploiting the separative powers of ESI-IMS–MS, we first describe higher order oligomeric states populated by Aβ40, under conditions compatible both with ESI–MS and fibril formation (as judged by TEM), and elucidate their CCSs. Aβ40 was dissolved initially in 100% DMSO to remove any preformed aggregates and diluted 100-fold into 200 mM ammonium acetate buffer, pH 6.8 before centrifugation (13,000*g*, 4 °C, 10 min) to remove any larger order species that may persist. The distribution of soluble oligomeric species was analysed immediately (within 2 min, post centrifugation), using ESI-IMS–MS. The data obtained (Fig. 2a and b) showed that high order oligomers are formed within 2 min of dilution of Aβ40 into buffer, consistent with previous analyses [6], [9], [18]. Co-populated oligomers with the same *m*/*z* can be separated using IMS–MS, for example, the dimer^4+^ and trimer^6+^ ions (Fig. 2b). Multiple charge states, predominantly triply and quadruply charged, and different conformers, both compact and expanded, are observed for the Aβ40 monomer [6], [9], [18] (data not shown). Aβ40 oligomer CCSs were estimated from the ESI-IMS–MS arrival time distributions and compared with CCSs estimated for theoretical oligomer growth models including a fit assuming isotropic growth [8], a fit assuming globular oligomers based on the average density of a protein under similar conditions (0.44 Da/Å^3^) [28], and a model that assumes growth in a single dimension (linear growth) [8]. CCS determination suggests that Aβ40 oligomers ⩾ trimer in size adopt relatively extended conformations rather than spherical or isotropic growth conformations (Fig. 2c). Ultimately, long, straight fibrils typical of amyloid form (Fig. 2a inset).

### 3.2 Focused screen for the identification of novel inhibitors of amyloid formation

**Fig. 3:**
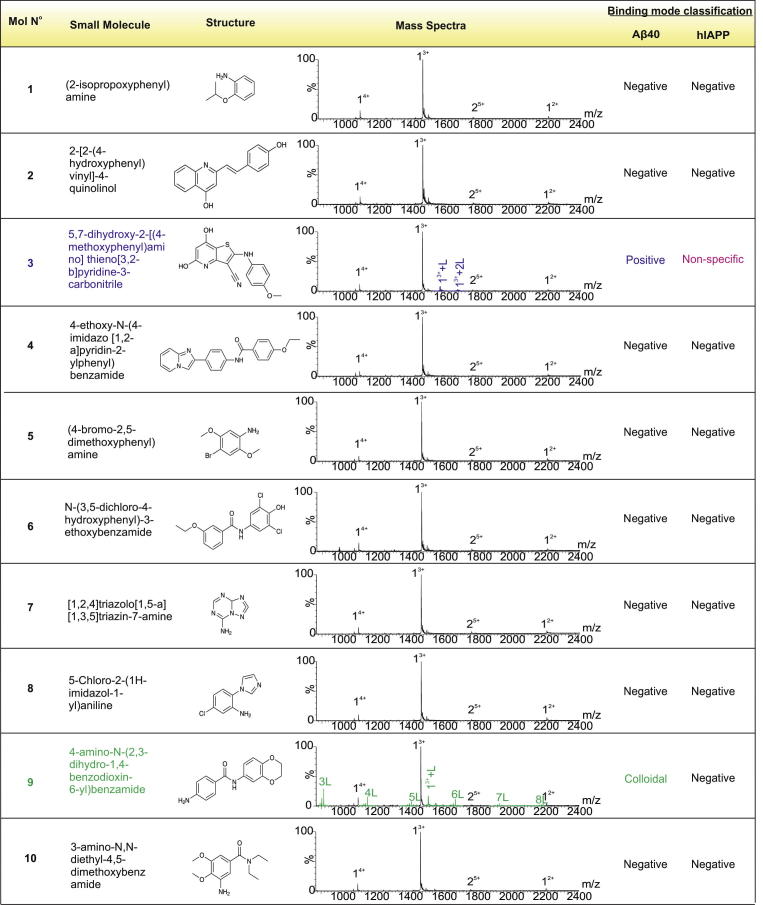

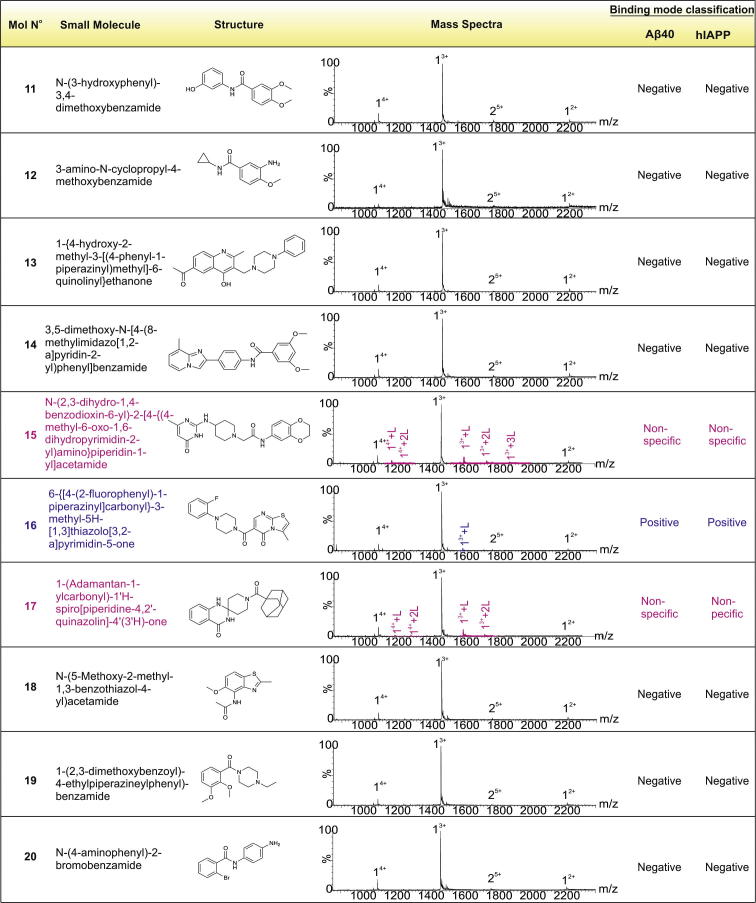
Focused high-throughput screen (HTS) results. Mass spectra labels indicate number of ligands (L) bound to each charge state of Aβ40. Binding modes as determined from the mass spectra are denoted as positive, negative, non-specific or colloidal. Molecule numbers **3** and **16** exhibit positive (specific) binding to Aβ40 (purple peaks); compounds **15** and **17** exhibit non-specific binding (pink peaks) and compound **9** exhibits colloidal binding (green peaks; each multimer of the small molecule is denoted nL, where *n* = oligomer number. The interaction of each small molecule with hIAPP is also shown [18].

Using the ESI-IMS–MS-based screening approach described above and in Young et al. [18], 20 compounds were selected from a library of novel molecules with structural similarity to five known inhibitors of Aβ40 aggregation previously reported. This focused screening method used the structural information from the known Aβ40 bioactive ligands *o*-vanillin [29], resveratrol [30], curcumin [31], chloronaphthoquinine-tryptophan (Cl-NQTrp) [32] and (−)-epigallocatechin gallate (EGCG) [5], [33] to identify novel compounds with related structural properties, but different chemistry and hence higher potential biological activity. This approach gives a higher hit-rate compared with random screening [34]. A subset of 20 compounds (Fig. 3) was chosen from a library of 50,000 lead-like small molecules for analysis using the comparator Rapid Overlay of Chemical Structures (ROCS) Combiscore [35], as described in [18]. These 20 compounds (named molecules 1–20) were added to monomeric Aβ40 and the binding mode of each was assessed by analysis of the resulting ESI-IMS–MS spectra. In parallel, the ability of the molecules to inhibit fibril formation was determined using negative stain TEM, after 5 days incubation at a 10:1 molar ratio of small molecule to Aβ40.

**Fig. 4:**
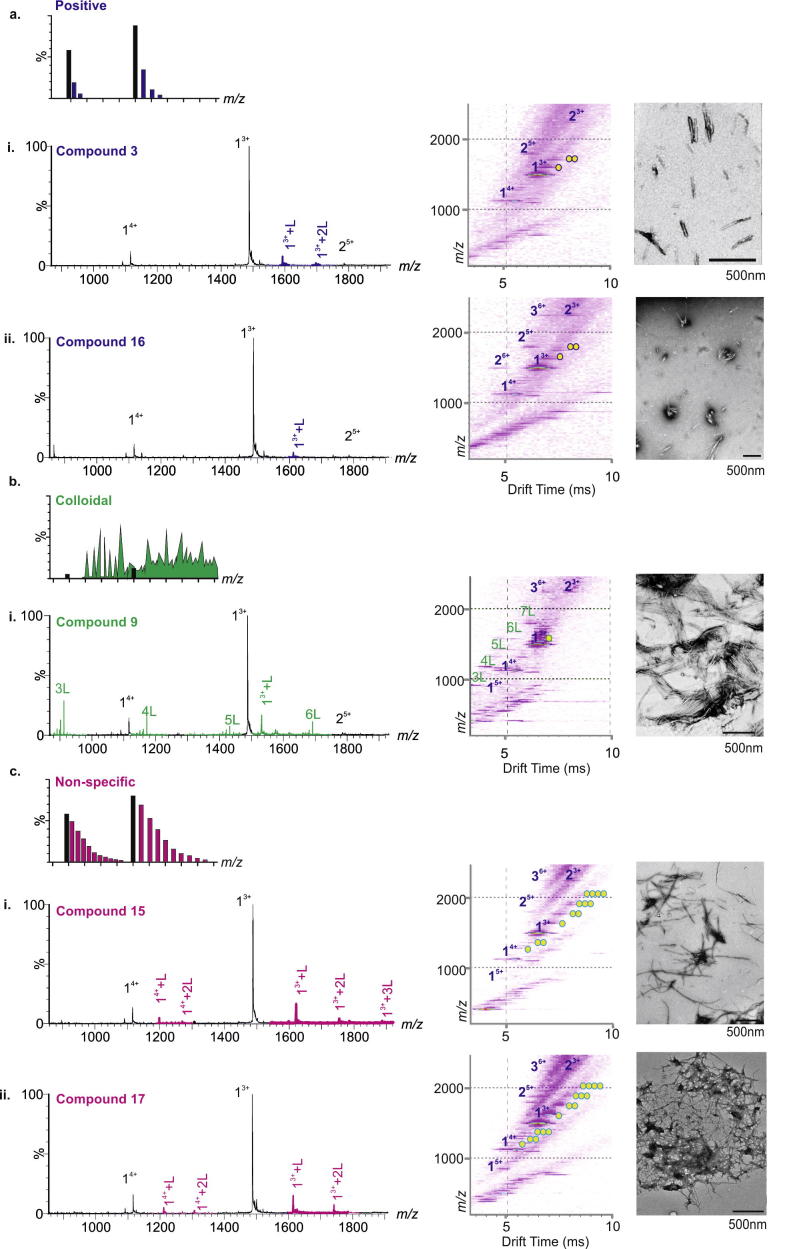
Positive, colloidal and non-specific binding molecules from focused HTS. (a) Molecule numbers **3** (i) and **16** (ii) exhibit ‘positive’ (specific) binding to Aβ40 monomer (purple peaks) according to the ESI-IMS–MS classification system and negative stain TEM images of Aβ40 incubated with 10:1 molar ratios of molecule: Aβ40 for 5 days in 200 mM ammonium acetate buffer, pH 6.8 (25 °C, quiescent) show the absence of fibrils after incubation. (b) Compound **9** (i) exhibits colloidal binding to itself (peaks denoted nL where n is the number of small molecules present in the aggregate) and to Aβ40 + nL (green peaks). (c) Compounds **15** (i) and **17** (ii) exhibit non-specific binding to Aβ40 (pink peaks). Compounds **9**, **15** and **17** fail to prevent fibrillation of Aβ40 (scale bar in nm is indicated at the foot of each TEM image). Circles in ESI-IMS–MS Driftscope images indicate the number of small molecules bound to each ion.

**Fig. 5:**
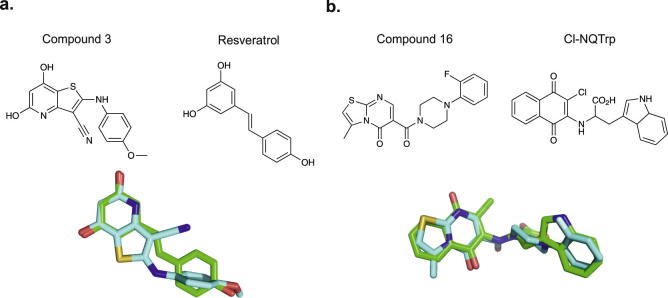
Structural comparison of Aβ40 inhibitors with their parent compounds in ROCS analysis. (a) Compound **3** (blue) and the parent molecule resveratrol (green). (b) Compound **16** (blue) and the parent molecule Cl-NQTrp (green).

The compounds were categorised according to the binding mode classification system described in Fig. 1 and in Young et al. [18]. A ‘positive’ small molecule that specifically interacts with the peptide will produce a binomial distribution of bound peaks. Conversely, a non-specific ligand will bind, but result in a Poisson distribution of bound peaks. A colloidal inhibitor will produce a range of overlapping peaks due to self-association of the small molecule and non-interacting ‘negative’ small molecules will not bind to the target peptide [18]. Of the 20 compounds screened, two were found to inhibit Aβ40 aggregation (compound **3** and compound **16**, Figs. 3 and 4a), one exhibited colloidal binding (compound **9**) (Figs. 3 and 4b) and two demonstrated non-specific binding, (compounds **15**, and **17**) (Figs. 3 and 4c). The remainder did not bind to Aβ40 (Fig. 3). Despite interacting with Aβ40, non-specific and colloidal binding of small molecules to target proteins is not useful therapeutically. The newly discovered inhibitors, compound **3** and compound **16** are structurally similar to, but chemically distinct from (as determined by ROCS Combiscore), the known inhibitors of Aβ40 aggregation, resveratrol [30] and Cl-NQTrp [32], respectively (Fig. 5). In the presence of a 10-fold molar excess of either compound, Aβ40 shows evidence of specific ligand binding and depletion of higher order oligomers such that only dimers and trimers are observed. Neither of the latter species is detected to bind the small molecule. TEM analysis confirms that fibril formation is inhibited and amorphous or short fibrillar aggregates accumulate (Fig. 4a). The low levels of binding observed for the ‘positive’ inhibitors, despite complete inhibition of fibrillation, are consistent with the fact that hydrophobic interactions are not wholly maintained in the gas-phase. Similar low levels of binding have been observed for ‘positive’ inhibitors of hIAPP, including EGCG [5], [18]. Gas-phase analysis of hydrophobic interactions between protein and ligands could lead to underestimation of binding affinity and/or false negative results [36]. For this reason, we would recommend that fibril formation is monitored over the time-course using ThT fluorescence and the morphologies of the resulting peptide aggregates are assessed using negative stain TEM, in addition to gas-phase analyses. Remarkably, 18 of the 20 lead compounds screened exhibit similar interactions with human islet amyloid polypeptide (hIAPP) [18] and Aβ40. The two exceptions are compound **3** (specific binding to Aβ40, non-specific binding to hIAPP [18]) and compound **9** (colloidal binding in the presence of Aβ40, no binding in the presence of hIAPP [18]). Interestingly, compound **16** binds specifically to both hIAPP and Aβ40, and inhibits fibrillation of both peptides *in vitro* [18]. Although hIAPP and Aβ40 share only 24% sequence identity and 46% sequence similarity, the core sequences of both, believed to key for self-assembly [37], [38], share 57% identity and 86% similarity (NNFGAIL in hIAPP, SNKGAII in Aβ40). The ability of compound **16** to interrupt the fibrillation process of both these peptides suggests that this small molecule may be either interacting directly with these comparable amyloidogenic sequences, or with an early oligomeric species with interaction interfaces common to both fibrillation pathways.

## 4 Conclusions

There is a pressing need both for a better understanding of the mechanisms of amyloid assembly and for new compounds able to halt the progression of amyloid diseases. Screening libraries of small molecule compounds and determining their mechanism of action is vital in the search for inhibitors of protein aggregation. The conventional screening method using Thioflavin T binding has resulted in several small molecules being erroneously described as inhibitors whereby, in fact, they were only inhibitors of the dye binding to the fibrils formed [39], [40], [41], [42]. Furthermore, many small molecules act in a promiscuous manner, binding non-specifically to many unrelated proteins [43]. This interaction, as well as colloidal binding, is undesirable in a proposed therapeutic [44], thus additional effort is required to eliminate these false-positive hits in conventional screens. ESI-IMS–MS enables the visualisation and quantification of oligomeric species populated early during amyloid formation, the specific species with which the small molecule binds and the consequences of binding on the course of aggregation [5], [17], [18], [20]. Identification of specific-binding at an early stage of screening efficiently rules out non-specific or colloidal ligands, providing leads for further analysis and development. A further advantage of the ESI-IMS–MS method described here (and in [18]) is that it is also amenable to high-throughput format, enabled by automation of the ESI-IMS–MS inlet and/or studying mixtures of small molecules in combination, as detailed elsewhere [18]. Although not employed in this instance, using robotic automation and assaying mixtures of 5 compounds within one sample, as described in Young et al. [18], would allow ∼5000 compounds to be screened in less than 24 h.

Here, we highlight the approach showing how, combined with ROCS Combiscore analysis, two new inhibitors of Aβ40 have been identified from 20 virtual hits. Combined with previous success in the discovery of new inhibitors of hIAPP aggregation [18], we envision that ESI-IMS–MS will play a pivotal role in future compound discovery in the anti-amyloid therapeutic field.

## Author contributions

L.M.Y. and J.C.S. contributed equally to this work. L.M.Y., J.C.S., S.E.R., and A.E.A. conceived and designed the experiments. L.M.Y., J.C.S. and R.A.M. performed the experiments; C.H.R. and R.J.F designed and prepared the screening library. L.M.Y. analysed the data. L.M.Y., J.C.S., R.A.M., C.H.R., R.J.F., A.E.A. and S.E.R. wrote the manuscript.

## References

[1] Sipe J.D., Benson M.D., Buxbaum J.N., Ikeda S.-I., Merlini G., Saraiva M.J.M., Westermark P. (2014). Amyloid.

[2] McKhann G.M., Knopman D.S., Chertkow H., Hyman B.T., Jack C.R., Kawas C.H., Klunk W.E., Koroshetz W.J., Manly J.J., Mayeux R., Mohs R.C., Morris J.C., Rossor M.N., Scheltens P., Carrillo M.C., Thies B., Weintraub S., Phelps C.H. (2011). Alzheimers Dement..

[3] Sakono M., Zako T. (2010). FEBS J..

[4] Hamley I.W. (2012). Chem. Rev..

[5] Young L.M., Cao P., Raleigh D.P., Ashcroft A.E., Radford S.E. (2014). J. Am. Chem. Soc..

[6] Kloniecki M., Jablonowska A., Poznanski J., Langridge J., Hughes C., Campuzano I., Giles K., Dadlez M. (2011). J. Mol. Biol..

[7] Gessel M.M., Bernstein S., Kemper M., Teplow D.B., Bowers M.T., Chem A.C.S. (2012). Neuroscience.

[8] Bleiholder C., Dupuis N.F., Wyttenbach T., Bowers M.T. (2011). Nat. Chem..

[9] Bernstein S.L., Dupuis N.F., Lazo N.D., Wyttenbach T., Condron M.M., Bitan G., Teplow D.B., Shea J.E., Ruotolo B.T., Robinson C.V., Bowers M.T. (2009). Nat. Chem..

[10] Ruotolo B.T., Benesch J.L., Sandercock A.M., Hyung S.J., Robinson C.V. (2008). Nat. Protoc..

[11] Smith D.P., Knapman T.W., Campuzano I., Malham R.W., Berryman J.T., Radford S.E., Ashcroft A.E. (2009). Eur. J. Mass Spectrom..

[12] Smith D.P., Radford S.E., Ashcroft A.E. (2010). Proc. Natl. Acad. Sci. U.S.A..

[13] Hernandez H., Robinson C.V. (2007). Nat. Protoc..

[14] Dupuis N.F., Wu C., Shea J.E., Bowers M.T. (2009). J. Am. Chem. Soc..

[15] Smith D.P., Woods L.A., Radford S.E., Ashcroft A.E. (2011). Biophys. J..

[16] Woods L.A., Radford S.E., Ashcroft A.E. (2013). Biochim. Biophys. Acta.

[17] Woods L.A., Platt G.W., Hellewell A.L., Hewitt E.W., Homans S.W., Ashcroft A.E., Radford S.E. (2011). Nat. Chem. Biol..

[18] Young L.M., Saunders J.C., Mahood R.A., Revill C.H., Foster R.J., Tu L.H., Raleigh D.P., Radford S.E., Ashcroft A.E. (2015). Nat. Chem..

[19] Susa A.C., Wu C., Bernstein S.L., Dupuis N.F., Wang H., Raleigh D.P., Shea J.-E., Bowers M.T. (2014). J. Am. Chem. Soc..

[20] McCammon M.G., Scott D.J., Keetch C.A., Greene L.H., Purkey H.E., Petrassi H.M., Kelly J.W., Robinson C.V. (2002). Structure.

[21] Hamrang Z., Rattray N.J.W., Pluen A. (2013). Trends Biotechnol..

[22] Walsh D.M., Thulin E., Minogue A.M., Gustavsson N., Pang E., Teplow D.B., Linse S. (2009). FEBS J..

[23] Finder V.H., Vodopivec I., Nitsch R.M., Glockshuber R. (2010). J. Mol. Biol..

[24] Giles K., Pringle S.D., Worthington K.R., Little D., Wildgoose J.L., Bateman R.H. (2004). Rapid Commun. Mass Spectrom..

[25] Valentine S.J., Counterman A.E., Clemmer D.E. (1999). J. Am. Soc. Mass Spectrom..

[26] Knapman T.W., Morton V.L., Stonehouse N.J., Stockley P.G., Ashcroft A.E. (2010). Rapid Commun. Mass Spectrom..

[27] Platt G.W., Routledge K.E., Homans S.W., Radford S.E. (2008). J. Mol. Biol..

[28] Lorenzen K., Olia A.S., Uetrecht C., Cingolani G., Heck A.J. (2008). J. Mol. Biol..

[29] De Felice F.G., Viera M.N.N., Saraiva L.M., Figueroa-Villar J.D., Garcia-Abreu J., Liu R., Chang L., Klein W.L., Ferreira S.T. (2004). FASEB J..

[30] Ladiwala A.R.A., Lin J.C., Bale S.S., Marcelino-Cruz A.M., Bhattacharya M., Dordick J.S., Tessier P.M. (2010). J. Biol. Chem..

[31] Yang F., Lim G.P., Begum A.N., Ubeda O.J., Simmons M.R., Ambegaokar S.S., Chen P.P., Kayed R., Glabe C.G., Frautschy S.A., Cole G.M. (2005). J. Biol. Chem..

[32] Scherzer-Attali R., Pellarin R., Convertino M., Frydman-Marom A., Egoz-Matia N., Peled S., Levy-Sakin M., Shalev D.E., Caflisch A., Gazit E., Segal D. (2010). PLoS One.

[33] Porat Y., Abramowitz A., Gazit E. (2006). Chem. Biol. Drug Des..

[34] Valler M.J., Green D. (2000). Drug Discov. Today.

[35] OEChem, version 1.7.4, OpenEye Scientific Software, Inc., Santa Fe, NM, USA, 2010. Available from: <http://www.eyesopen.com>.

[36] Wang W., Kitova E.N., Klassen J.S. (2003). Anal. Chem..

[37] Tenidis K., Waldner M., Bernhagen J., Fischle W., Bergmann M., Weber M., Merkle M.-L., Voelter W., Brunner H., Kapurniotu A. (2000). J. Mol. Biol..

[38] Colletier J.-P., Laganowsky A., Landau M., Zhao M., Soriaga A.B., Goldschmidt L., Flot D., Cascio D., Sawaya M.R., Eisenberg D. (2011). Proc. Natl. Acad. Sci..

[39] Aitken J.F., Loomes K.M., Konarkowska B., Cooper G.J.S. (2003). Biochem. J..

[40] Wu C., Lei H., Wang Z., Zhang W., Duan Y. (2006). Biophys. J..

[41] Noor H., Cao P., Raleigh D.P. (2012). Protein Sci..

[42] Meng F., Marek P., Potter K.J., Verchere C.B., Raleigh D.P. (2008). Biochemistry.

[43] Arkin M.R., Wells J.A. (2004). Nat. Rev. Drug Discov..

[44] Feng B.Y., Toyama B.H., Wille H., Colby D.W., Collins S.R., May B.C.H., Prusiner S.B., Weissman J., Shoichet B.K. (2008). Nat. Chem. Biol..

[45] Daubenfeld T., Bouin A.-P., van der Rest G. (2006). J. Am. Chem. Soc..

